# One surgeon’s experience with r-TAPP: a retrospective analysis of 150 consecutive robotic inguinal hernia cases

**DOI:** 10.1007/s11701-021-01336-y

**Published:** 2022-01-08

**Authors:** Jorge L. Florin, Valeria Bianchi, Daniel D. Wiggan

**Affiliations:** 1 Mid-Florida Surgical Associates, Ocoee, USA; 2 University of Central Florida, College of Medicine, 2799 Valeria Rose Way, Ocoee, FL 34761 USA

**Keywords:** Robotic hernia, Inguinal hernia, Hernia surgery, r-TAPP, Hernia repair

## Abstract

There is a tremendous paucity of literatures regarding the long-term surgical outcomes of the r-TAPP procedure for inguinal hernia repair. Additionally, much of the existing literatures regarding this procedure have limited follow-up of to 12 months. This article presents the outcomes of 150 consecutive r-TAPP inguinal hernia repairs performed on 111 patients using Progrip mesh without fixation, with up to 24 months of follow-up. The initial 150 consecutive r-TAPP inguinal hernia repairs were performed from February 2017 to April 2018 using Progrip without fixation. All patients were seen at 2 weeks, followed by phone follow-up at 6 months, 1 year, and 2 years. Of the 111 patients, 39 had bilateral hernias (35%) and 72 had unilateral hernias (65%). The age range was 18–93 years. The BMI range was 20.7–50.2, with a mean of 26.4 and median of 25.8. Total operative time ranged from 28 to 138 min with a mean of 62.4 min and median of 56 min. ASA classification ranged from 1 to 4, with a mean of 2.1. No significant blood loss was observed in any of the cases. There were no conversions to open surgery. All patients were discharged the same day of the operation. We were able to follow up with 100% of the hernias at 2 weeks, 88% at 6 months, 87% at 1 year, and 80% at 2 years. No recurrences were recorded at 2 weeks, 3 months, 6 months, 1 year, or 2 years. There were no reports of chronic pain up to 2 years in any of the patients. These results indicate that r-TAPP inguinal hernia repair using Progrip without further fixation is safe, effective, and can be performed with minimal recurrences or chronic pain.

**Supplementary Information:**

The online version contains supplementary material available at 10.1007/s11701-021-01336-y.

## Background

The surgical repair of inguinal hernia defects has evolved in parallel with the exponential growth of surgery as an academic discipline, catalyzed by the advent of anesthesia and aseptic technique in the mid-1800s [1]. Complication rates significantly decreased in the wake of this development; however, long-term integrity of inguinal hernia repairs remained unobtainable, with recurrence rates at 100% at 4-year follow-up [2]. While the Bassini, McVay and Shouldice methods of primary tissue repair serve as an operative tool in the setting of contamination where mesh placement is contraindicated [3], open tension-free repair using synthetic mesh as pioneered by Lichtenstein provided a robust framework for the development of modern techniques. Over 1000 cases using this technique were published, showing no recurrences at 5 years [2]. The modern era of minimally invasive inguinal hernia repair began with the introduction of laparoscopic techniques, namely trans-abdominal pre-peritoneal repair (TAPP) and totally extraperitoneal repair (TEP). These minimally invasive techniques allowed for a decrease in postoperative pain and earlier return to normal activities.

Presently, the use of robotic assistance in these procedures (r-TAPP and r-eTEP, respectively) enables greater intraoperative mobility when performing wristed movements compared to laparoscopic TAPP and TEP repair, and in this surgeon’s opinion, lends itself to a more complete dissection of the myopectineal orifice. Robotic assistance also mediates tremors and provides improved visualization of the operative field. Indeed, the rate of complications is greatly reduced relative to open repair (2.7% compared with 11.2% in one study) [4].

There is a tremendous paucity of literature regarding the long-term surgical outcomes of the r-TAPP procedure for inguinal hernia repair. Additionally, much of the existing literatures (including literature with large sample sizes) regarding this procedure have very limited follow-up of up to 12 months [5]. This article seeks to bridge this gap in the existing hernia literature by presenting the outcomes of 150 consecutive r-TAPP inguinal hernia repairs performed with the Intuitive Da Vinci Xi Surgical Robot on 111 patients using Progrip mesh without fixation, with up to 24-month follow-up.

## Methods

The initial 150 consecutive r-TAPP inguinal hernia repairs were performed with the Intuitive Da Vinci Xi Surgical Robot from February 2017 to April 2018 using Progrip without fixation. All patients were seen at 2 weeks, followed by phone follow-up at 6 months, 1 year, and 2 years. During the follow-up calls, we inquired about chronic pain or symptoms of hernia recurrence. Those that had any concerns were asked to make a follow-up appointment for further evaluation.

## Procedure

Three ports are placed in the epigastrium. One is placed in the left upper quadrant using a 5 mm Opti-view trochar, the second in the subxiphoid area, and the third in the right upper quadrant, the last two under direct visualization. The 5 mm Opti-view is then changed over to a robotic port under direct visualization. The patient is placed in Trendelenburg position and the robot is docked. The retrorectus space on the side of the hernia is entered 8 cm above the hernia defect and the line of dissection is carried laterally. As the midpoint of the rectus muscle is approached, the dissection is carried more superficially and the pre-peritoneal space is entered. The medial dissection is then carried to the symphysis pubis. Laterally, the space of Bogros is dissected out. In the middle, the hernia sac is reduced. If present, the cord lipoma is removed. The inferior and medial dissection is the most critical. The dissection proceeds 2 cm inferior to the symphysis and 2 cm across the midline. A 12 by 16 cm Progrip mesh covering the entire myopectineal orifice is placed, extending across the midline by 2 cm and inferior to the symphysis by 2 cm. The peritoneum is closed with a running 2–0 V-lock suture. CO_2_ is suctioned out of the pre-peritoneal space and the mesh can be seen lying flat.

## Results

Of the 111 patients involved in this case study, 39 had bilateral hernias (35%) and 72 had unilateral hernias (65%). 58% of the patients were classified as having an indirect inguinal hernia, 25% as having a direct inguinal hernia, and 16% as having both direct and indirect inguinal hernias. The age range for all patients was 18–93 years, with the largest age bracket (32.4%) being comprised of patients aged 66–77 years. The BMI range for all patients was 20.7–50.2, with a mean of 26.4 and median of 25.8. Total operative time ranged from 28 to 138 min with a mean of 62.4 min and median of 56 min. The mean operative times for left-sided, right-sided, and bilateral inguinal hernias were 55 min, 53 min, and 78 min, respectively. The majority of patients’ operative times (66 patients) fell within the range of 28 min to 58 min. ASA classification ranged from 1 to 4, with a mean of 2.1. No significant blood loss was observed in any of the cases. There were no conversions to open surgery. All patients were discharged the same day of the operation. All patients were classified as Grade I on the Clavien–Dindo scale for surgical complications. Of the 150 hernias, we were able to follow up with 100% at 2 weeks, 88% at 6 months, 87% at 1 year, and 80% at 2 years. Two patients were deceased at 2-year follow-up. No recurrences were recorded at 2 weeks, 6 months, 1 year, or 2 years. There were no reports of chronic pain up to 2 years in any of the patients. These data are illustrated in Tables 1, 2, 3, 4, 5, 6, 7, 8, 9.

**Table 1:** Hernia follow-up percentage at 2 weeks, 6 months, 1 year, and 2 years

Follow-up interval	Response %
2 weeks	100
6 months	88
1 year	87
2 years	80

**Table 2:** Low, high, mean and median values for patient age, BMI, ASA classification, and operative time

	Age (years)	BMI (kg/m^2^)	ASA	OR time (mins)
Low	18	20.7	1	28
High	93	50.2	4	138
Mean	59.6	26.4	2.1	62.4
Median	62.5	25.8	2	56

**Table 3:** Laterality represented as a percentage of patient sample

Laterality	Number of patients	% of patients
Unilateral	72	65
Bilateral	39	35

**Table 4:** Clavien–Dindo classification of surgical complications

Grade	Description
1	Any deviation from the normal postoperative course without the need for pharmacological treatment or surgical, endoscopic and radiological interventions This grade also includes wound infections opened at the bedside
2	Requiring pharmacological treatment with drugs other than such allowed for grade I complications. Blood transfusions, antibiotics and total parenteral nutrition are also included
3	Requiring surgical, endoscopic or radiological intervention
3a	Intervention under regional/local anesthesia
3b	Intervention under general anesthesia
4	Life-threatening complication requiring intensive care/intensive care unit management
4a	Single organ dysfunction
4b	Multi-organ dysfunction
5	Patient demise

**Table 5:** Breakdown of patient cohort by operative time interval

Operative Time Interval (minutes)	Number of patients
[28, 43]	17
[43, 58]	46
[58, 73]	16
[73, 88]	19
[88, 103]	7
[103, 118]	4
[118, 133]	1
[133, 148]	1

**Table 6:** Breakdown of mean operative times by hernia laterality

Laterality	Mean time (minutes)
Left	55
Right	53
Bilateral	78

**Table 7:** Patient follow-up progression

Time interval	No recurrence	Left message	Unable to contact	Deceased
2 weeks	111	0	0	0
6 months	98	12	1	0
1 year	96	14	1	0
2 years	89	10	10	2

**Table 8:** Breakdown of patient cohort by hernia classification

Classification	% of population
Direct	25
Indirect	58
Direct + indirect	16
Unspecified	1

**Table 9:** Patient age group distribution

Age	No. patients
[22, 33]	3
[33, 44]	10
[44, 55]	15
[55, 66]	27
[66, 77]	36
[77, 88]	18
[88, 99]	2

## Discussion

Primary concerns cited in the literature include a steep learning curve, cost, and an increase in OR time compared to L-TAPP or TEP. However, the steepness of the learning curve is greatly reduced for surgeons accustomed to performing laparoscopic repairs, given the similarity to the robotic procedure with few adjustments [4]. This is evidenced by the study by Kudzi et al. showing identical OR times despite a higher number of complex cases being performed in the r-TAPP group [6]. This sentiment is further supported by the study performed by Kakiashvili et al. showing the same median OR time for the laparoscopic and robotic procedures. Our study shows similar OR times relative to the laparoscopic procedure with similar laterality. In our study, OR times are seen to decrease with experience.

Although short-term outcomes are relatively well-represented in the literature, further generation of data demonstrating the incidence of chronic pain or recurrence at long-term follow-up is warranted. The incidence of chronic pain is decreased via use of the robot, likely in part due to the increased dexterity and visualization as well as eliminating the use of mesh fixation.

The recurrence rate in the Kakiashvili study was 8.3%, which is higher than that of the open and laparoscopic rates in the same study (3.1% and 0%) [2]. Another study analyzing short-term outcomes from 22 surgeons using the TEP procedure found a 0.8 percent recurrence rate at 3 months with a sample size of 665 patients [7]. However, our study shows outcomes from a single surgeon using a large sample size of 150 hernias with zero recurrences at 2-year follow-up.

The primary limitation of our study is that it is a case series describing outcomes of procedures performed by a single surgeon. More data by other surgeons would further substantiate the viability of r-TAPP as a technically feasible method of hernia repair, of which widespread adoption could be warranted provided its economic feasibility were to be accounted for.

Another limitation of our study is the timeframe over which the study was performed. Although our follow-up length of 2 years represents a substantial improvement over follow-up in the current hernia literature, it should be noted that in the current instantiation of our study, it does not account for very long-term follow-up (> 10 years). A chart review performed by Barbaro et al. during the early inception of TEP analyzed recurrence rates over a 20-year period. This review demonstrated that 33% of TEP recurrences occurred after a period of > 10 years [8]. Advancements in TEP have improved recurrence rates considerably since its inception, but it remains unclear whether these improvements will translate to improved very long-term recurrence rates. Our study shows zero recurrences with the Da Vinci platform at 2-year follow-up but is presently unable to account for recurrence rates in the very long term. We are currently performing a longer-term study with up to 5-year follow-up.

With regard to mesh selection, the decision to use Progrip mesh was based on it being a lightweight, monofilament, macro-porous mesh. Additionally, fixation has been shown to lead to postoperative pain. Progrip mesh is self-gripping, so there is no need for fixation. In our study, no mesh migration was apparent since there were no reports of recurrence or chronic pain.

Taken together, these data show that, provided the learning curve is navigated successfully, excellent results can be achieved without increased operative times or the need for mesh fixation.

## Conclusion

These results indicate that r-TAPP inguinal hernia repair using Progrip without further fixation can be performed with similar OR times as laparoscopic repairs. In addition, it appears to be both safe and effective, and can be performed with minimal recurrences or chronic pain.

## Supplementary Information

Below is the link to the electronic supplementary material.

Supplementary file1 (MOV 156052 KB)
